# Adverse childhood experiences and adult inflammation: Single adversity, cumulative risk and latent class approaches

**DOI:** 10.1016/j.bbi.2020.03.017

**Published:** 2020-07

**Authors:** Rebecca E. Lacey, Snehal M. Pinto Pereira, Leah Li, Andrea Danese

**Affiliations:** a Research Department of Epidemiology and Public Health, University College London, 1-19 Torrington Place, London WC1E 6BT, United Kingdom; b Population, Policy and Practice Research and Teaching Department, University College London Great Ormond Street Institute of Child Health, London, 30 Guilford Street, WC1N 1EH, United Kingdom; c Social, Genetic and Developmental Psychiatry Centre and Department of Child and Adolescent Psychiatry, Institute of Psychiatry, Psychology and Neuroscience, King’s College London, De Crespigny Park, London SE5 8AF, United Kingdom; d National and Specialist CAMHS Clinic for Trauma, Anxiety, and Depression, South London and Maudesley NHS Foundation Trust, De Crespigny Park, London SE5 8AZ, United Kingdom

**Keywords:** Adverse childhood experiences, Birth cohort, Cumulative risk, Inflammation, Latent class analysis, National Child Development Study

## Abstract

**Background:**

Adverse childhood experiences (ACEs) have long been known to be related to poorer health across the life course. Previous studies typically relied on cumulative risk scores or individual adversities measured through retrospective self-reports. However, these approaches have important limitations. Cumulative risk scores assume equal weighting of adversities and the single adversity approach ignores the high probability that adversities co-occur. In contrast, latent class analysis (LCA) offers an alternative approach to operationalise ACEs that respects the clustering of adversities and may identify specific patterns of ACEs important for health outcomes. Furthermore, prospective and retrospective reports of ACEs show poor agreement. Therefore, it is important to compare findings based on prospective and retrospective measures in the same individuals. Despite an increasing number of studies applying LCA to ACEs data, no studies have yet simultaneously investigated LCA to cumulative risk and single adversity approaches in their relationships with adult inflammation. Identifying the specific ACEs or combinations of ACEs which are strongly related to inflammation is important for investigating the mechanisms involved and the planning of effective interventions.

**Methods:**

Using data on 8810 members of the 1958 British birth cohort we investigated 12 ACEs – physical, psychological and sexual abuse, physical and emotional neglect, parental mental health problems, witnessing abuse, parental conflict, parental divorce, parental offending, parental substance misuse and parental death. LCA was applied to explore the clustering of prospectively and retrospectively reported ACEs separately. Associations between latent classes, cumulative risk scores and individual adversities with three inflammatory markers (C-Reactive Protein, fibrinogen and von Willebrand Factor) were tested using linear regression.

**Results:**

There was co-occurrence between adversities, and particularly for retrospectively reported adversities. Three latent classes were identified in the prospective data – ‘Low ACEs’ (95.7%), ‘Household dysfunction’ (2.8%) and ‘Parental loss’ (1.5%) which were related to increased inflammation in mid-life, as were high ACE scores and individual measures of offending, death, divorce, physical neglect and family conflict. Four latent classes were identified in the retrospective data – ‘Low ACEs’, ‘Parental mental health and substance misuse’, ‘Maltreatment and conflict’ and ‘Polyadversity.’ The latter two (5.2%) were related to raised inflammation in mid-life, as was a retrospective ACE score of 4+ (8.3%) and individual measures of family conflict, psychological and physical abuse, emotional neglect and witnessing abuse.

**Conclusions:**

Specific ACEs or ACE combinations might be important for chronic inflammation. LCA is an alternative approach to operationalising ACEs data but further research is needed.

## Highlights

• There is a reliance on ACE scores and single adversity approaches in ACEs research.
• Latent class analysis (LCA) offers an alternative approach to operationalising ACEs.
• We apply LCA to a large longitudinal study with prospective and retrospective ACEs data.
• We compare LCA to ACE scores and individual ACE approaches in relations with inflammation.
• Specific ACEs and ACE combinations are important for inflammation in mid-life.

## 1 Introduction

### 1.1 Adverse childhood experiences and inflammation

The relationship between adverse childhood experiences (ACEs), such as child maltreatment, parental divorce and parental mental illness, and a wide range of poorer health outcomes has been extensively studied, e.g.(Bellis et al., 2019). The underlying biological mechanisms linking ACEs and poorer health are being increasingly unravelled thanks to the growing availability of high-quality longitudinal datasets with information on both ACEs and biomarkers. One particularly salient biological pathway of interest involves chronic inflammation. Inflammation forms part of the innate immune response to physical trauma and infection. However, chronic activation of the inflammatory response can be harmful and is thought to be one of the key biological mechanisms linking ACEs to psychopathology (Danese and Baldwin, 2017) and cardiometabolic disease (Baldwin and Danese, 2019).

ACEs have been linked to chronic inflammation across the life course. For instance, recent studies have shown that early life adversities, such as parental mental illness (O’Connor et al., 2019) and the number of adversities experienced prior to age 9 (Flouri et al., 2020, Slopen et al., 2013) were associated with elevated Interleukin-6 (IL-6) and C-Reactive Protein (CRP) levels in childhood and adolescence. Also children exposed to multiple ACEs or maltreatment in childhood had higher CRP levels in early adulthood (Baldwin et al., 2018, Danese et al., 2007) and beyond (Chen and Lacey, 2018). A systematic review by Baumeister et al (2015) included 25 studies, finding that overall childhood trauma was linked with higher levels of inflammation in adulthood.

### 1.2 Operationalising ACEs

#### 1.2.1 ACE scores and single adversity approaches

There has been little consideration in the ACEs and inflammation literature thus far on how ACEs are operationalised and in comparing different methods. This is important to consider in order to elucidate the underlying mechanisms and planning of effective interventions. It is recognised that ACEs tend to cluster so that people reporting one adversity are more likely to report others. In the Kaiser Permanente ACE study a high proportion of participants (between 81 and 98%) reporting an adversity reported at least one other (Dong et al., 2004). Generally, studies into the health effects of ACEs have relied on a simple approach using cumulative adversity (i.e. ACE scores) whereby the number of adversities reported are summed to deal with this ACE clustering. For instance, Felitti’s study of adults in the Kaiser Permanente Adverse Childhood Experiences Study demonstrated a graded relationship between retrospectively reported ACE scores and multiple negative health outcomes, including risky health behaviours, heart disease, cancer and lung conditions (Felitti et al., 1998).

The ACE score approach has been widely applied in ACEs and health research, including to studies of inflammation (Chen and Lacey, 2018, Rasmussen et al., 2019, Slopen et al., 2015). However there are several limitations of this approach when investigating associations with health (Lacey and Minnis, 2019). The most notable of these are the assumption that each adversity is equally important for a specific health outcome and the specific patterning of ACEs co-occurrence is ignored. For instance, the combination of parental mental illness and parental separation (ACE score of 2) is treated as the same as physical and sexual abuse (also an ACE score of 2) – which is unlikely to be the case. Moreover, as discussed elsewhere (Danese, 2019, Kelly-Irving and Delpierre, 2019), there are concerns regarding the potential misuse of ACE questionnaires and the resulting score.

One alternative to the ACE score approach is to explore the effect of each adversity separately. There are numerous studies which examine the association of single adversities on inflammation, e.g.(Pinto Pereira et al., 2019). It is likely that different adversities show different associations with inflammation. In a meta-analysis of childhood maltreatment and adult inflammation, sexual and physical abuse were associated with higher Tumour Necrosis Factor-α (TNF-α) and IL-6 but psychological abuse was not (Baumeister et al., 2015). Also parental absence was associated with raised CRP but abuse experiences were not. Whilst looking at single adversities might be informative in teasing out the life course mechanisms involved e.g. (Lacey et al., 2013), the effects of that adversity might be confounded by the experience of other adversities which have not been accounted for in the analyses.

#### 1.2.2 Person-centred approaches

Given the increasing recognition of the limitations of ACE score and single adversity approaches in ACEs research, there has been an emergence of alternative methods of operationalising ACEs which respect their clustering and are more informative when looking at associations with health. These alternative analytic approaches have included the person-centred methods (e.g. Latent Class Analysis, LCA). LCA is a data-driven approach which aims to identify distinct groups or classes of individuals who have similar patterns of reported adversities (Masyn, 2013). The use of LCA with ACEs data has increased in the past few years and allows the researcher to explore whether the specific patterning of ACEs is important for health outcomes.

Several studies have explored whether ACE classes obtained using LCA show differing associations with health outcomes. In a sample of German children aged 4–17 years, Witt et al. (2016) identified three maltreatment classes using LCA – ‘Multiple types of maltreatment excluding sexual abuse’ (63.1%), ‘Multiple types of maltreatment including sexual abuse’ (26.5%) and ‘Predominately sexual abuse (10.3%)’. The second class showed the poorest health outcomes in terms of mental disorders and health-related quality of life suggesting that the combination of sexual abuse with other maltreatments (physical and psychological abuse, witnessing domestic violence and neglect) was particularly detrimental to health. Ho et al (2019) also identified three ACE classes among university students in Hong Kong – ‘Low ACEs’ (76.0%), ‘Household violence’ (20.6%) and ‘Household dysfunction’ (3.4%). Students in the ‘Household violence’ class – characterised by high probability of reporting physical and psychological abuse and domestic violence - were more likely to report depression and maladjustment symptoms than those in the ‘Low ACEs’ class.

Whilst LCA is showing promise as an emerging approach to operationalising ACEs data, few studies have applied LCA to adversities beyond child maltreatment and there have been no studies to the authors’ knowledge that have explored whether different LCA-derived clusters show different associations with inflammation.

#### 1.2.3 Comparing methods of ACEs operationalisation

There are few studies comparing the main approaches – ACE scores, single adversities and LCA - to ACEs operationalisation. Lanier et al. (2018) compared LCA to ACE scores using the 2011/12 US National Survey of Children’s Health. The ACE score showed a graded relationship with poor child health, the presence of chronic health conditions and special healthcare need – the three child health outcomes in the study. The LCA identified seven classes ‘0–1 ACEs’ (76%), ‘1–2 ACEs’ (11%), ‘Domestic violence, no mental illness’ (3%), ‘Mental illness and poverty’ (1%), ‘Substance use and incarceration’ (2%), and ‘High ACEs’ (2%). The ‘Mental illness and poverty’ class was most strongly associated with poorer child health outcomes, suggesting that this specific ACE combination might be more important for the child health.

Similarly, Merians et al (2018) compared ACE scores to LCA of retrospectively-reported ACE measures from a sample of US college students. The differences between students with an ACE score of 5+ and those with no ACEs were comparable to the difference between the ‘High ACEs’ and ‘Low ACEs’ classes identified from the LCA, in terms of the magnitude of association and also the variance explained in the three outcomes (physical health, alcohol use consequences and academic performance). Whilst these two studies compared two approaches to ACE operationalisation – typically ACE scores and LCA – there have been no studies as yet which have compared ACE scores, LCA and single adversities. Further there have been no studies which have investigated how different ACE operationalisations are linked to inflammation in adulthood.

#### 1.2.4 Prospective vs retrospective ACEs measures

Another key methodological consideration in ACEs research is whether the information on adversities is reported prospectively or retrospectively. There has been a large reliance thus far on retrospective reports of adversities experienced in childhood. Recent work has demonstrated poor agreement between prospective and retrospective ACE reports, with the exception of separation from a parent (Baldwin et al., 2019). Retrospective ACEs reports are influenced by various factors such as personality, current mental wellbeing and life stress (Colman et al., 2015, Jaffee, 2017, Reuben et al., 2016). Given these findings it is important that prospective and retrospective information is not used interchangeably.

### 1.3 Gaps in the literature and aim of the present study

Several gaps have been identified in the current literature. There have been no studies as yet which have compared LCA, ACE scores and single adversity approaches to ACEs with adult inflammation. This is essential to investigate because it is likely that specific ACEs or ACE combinations are important for inflammatory responses to adversity. Furthermore, prospective and retrospective reports of ACEs show poor agreement. Therefore, it is important to directly compare findings based on prospective and retrospective measures in the same individuals. Given these gaps, the aim of the present study was to examine the strength of associations between three approaches to ACEs operationalisation – ACE scores, single adversities and LCA – and two types of ACEs measures – prospective and retrospective - for adult inflammation in a large, longitudinal dataset in Great Britain (the 1958 British birth cohort).

## 2 Material and methods

### 2.1 Sample

This study used data from the National Child Development Study (the 1958 British birth cohort) which aimed to recruit all babies born during a single week of 1958 (achieved sample n = 17,415, 98.2% of all births that week) in Great Britain (Power and Elliott, 2005). There have been 11 waves of data collection so far – at birth, ages 7, 11, 16, 23, 33, 42, 44/45, 46, 50 and 55 years of age. The study sample was augmented in waves two to four (ages 7, 11 and 16) to include children born overseas in the relevant week who moved to Great Britain (n = 173, 202 and 295 respectively) (Johnson and Brown, 2015). The surveys included varied topics such as health, education, employment, social relationships and socioeconomic factors. The follow-up at age 44/45 included a biomedical survey on a sub-sample of cohort members (n = 9377, 77.9% of target), during which blood samples were taken allowing for the assessment of systemic inflammation. Given that inflammation is the outcome of the present study, we use data from birth through to age 44/45. Ethical approval for each follow-up survey was obtained from a UK Multi-centre Research Ethics Committee (Shepherd, 2012) and informed consent obtained from cohort members at different ages.

### 2.2 Adverse childhood experiences

The NCDS has both prospectively and retrospectively measured ACEs data. Prospectively measured ACEs were obtained from parental interviews (usually the mother), health visitor or teacher assessment at ages 7, 11 and 16. The prospectively measured adversities were physical neglect, parental mental health problems, parental conflict, parental separation/divorce, parental offending, parental substance misuse and parental death.

Regarding retrospective measures, cohort members were also asked about parental divorce during childhood when they were age 33. At age 44/45 cohort members were asked to report a range of adverse experiences in childhood (up to age 16) using a confidential computer-based questionnaire. These included physical, psychological and sexual abuse, emotional neglect, parental mental health problems, witnessing abuse, family conflict and parental substance misuse. Given the poor agreement between prospective and retrospectively measured ACEs (Baldwin et al., 2019, Newbury et al., 2018) we did not combine the prospectively and retrospectively measured data, but instead considered these as two separate groups of variables for all analyses. Further information on prospective and retrospective ACE reports used and their collection are shown in Supplement 1.

### 2.3 Adult inflammation

Three inflammatory markers were measured from blood samples collected at age 44/45 years. CRP was measured on citrated plasma by high-sensitivity nephelometric analysis of latex particles with CRP-monoclonal antibodies (Elliott et al., 2008). Cohort members with CRP values ≥ 10 mg/L indicative of recent infection or trauma were excluded from the analyses (see Section 2.5.3 below). Fibrinogen (g/L) was also measured on citrated plasma by the Clauss method. Von Willebrand Factor (vWF, IU, dl) was measured on citrated plasma by enzyme-linked immunosorbent assays using a double sandwich technique (Elliott et al., 2008). Whilst all three markers play a role in inflammatory pathways, fibrinogen and vWF also have endothelial and clotting functions. vWF is less commonly used in epidemiological analyses but the role of vWF in inflammation is becoming increasingly recognised (Kawecki et al., 2017). All three inflammatory markers were positively skewed and hence log-transformed for analyses.

### 2.4 Covariates

The covariates included in our analyses were gender and two indicators of socioeconomic circumstances at birth - father’s occupational social class (measured using the Registrar General’s Social Class Schema as ‘I professional’, ‘II managerial and technical’, ‘IIINM skilled non-manual’, ‘IIIM skilled manual’, ‘IV semi-skilled manual’ or ‘V unskilled manual’) and maternal education (‘mother stayed in education beyond minimum age’ or ‘mother left at/before minimum age’). We also included variables indicating the health of the infant, including birthweight (grams), gestational age (days), maternal age at birth and breastfeeding duration (‘none’, ‘up to one month’ or ‘longer than one month’). These covariates have been used in prior work on this topic using this cohort (Chen and Lacey, 2018, Li et al., 2019, Pinto Pereira et al., 2017).

### 2.5 Statistical analysis

#### 2.5.1 Missing data

Missing data is an important source of bias in longitudinal studies, as cohort members who have missing information or drop out of longitudinal studies tend to be less healthy and more socially disadvantaged than those who do not (Allison, 2002). In order to minimise the bias attributable to missing information, missing data on covariates and prospectively measured ACEs were imputed using multiple imputation by chained equations, imputing information for cohort members who had at least one observed inflammatory marker and who had completed the retrospective ACEs questionnaire (n = 8810, 94.0% of those present in the biomedical survey at age 44/45). The imputation models included all analysis variables plus auxiliary variables predictive of missingness (e.g. repeated measures of housing tenure, overcrowding, financial hardship, occupational social class). Twenty imputed datasets were created and the estimates were combined across datasets using Rubin’s rules (Rubin, 1987).

#### 2.5.2 Operationalisation and clustering of adverse childhood experiences

As mentioned in Section 2.2, the prospective and retrospective ACEs were treated separately in our analyses. We first explored descriptively the clustering of ACEs by viewing the row percentages of those reporting each adversity against all other adversities. Additionally, we constructed a tetrachoric correlation matrix calculating Rho coefficients to indicate the strength of association between all adversities. Three methods for considering ACEs were then applied. Firstly, each ACE was considered individually. Secondly, we calculated the cumulative ACE score by summing the number of adversities reported. Based on the distribution of cohort members on this variable, the prospective ACE score was banded as ‘0 ACEs’, ‘1 ACE’ or ‘2 + ACEs’ (only 1.8% of cohort members reported 3 or more ACEs prospectively), and as ‘0 ACEs’, ‘1 ACE’, ‘2 ACEs’, ‘3 ACEs’ and ‘4 + ACEs’ for the retrospective ACE score. The latter had more categories with the intention of being consistent with much of the ACE score literature e.g. (Bellis et al., 2017, Felitti et al., 1998). Thirdly, to identify different ACE clusters we applied LCA using the robust maximum likelihood estimator. We compared models for 2 to 6 classes. The best fitting class solution was chosen based on model fit statistics – Bayesian Information Criteria (BIC), Akaike’s Information Criteria (AIC) and sample-size adjusted BIC (SSABIC). Lower values of the BIC, AIC and SSABIC indicate a better fitting model and an entropy value approaching 1 represents good distinction between classes (Celeux and Soromenho, 1996). In determining the most optimal class solution, preference was given to improvements in BIC and more specifically to the point beyond which there were diminishing returns in BIC improvement (Nylund-Gibson and Young Choi, 2018). Upon obtaining the best class solution, individuals were assigned to their most likely class, creating a categorical ACEs cluster variable. Latent profile plots were created for the final solutions to aid interpretation of the quantitative and qualitative differences between clusters (Debowska et al., 2017).

#### 2.5.3 Associations between adverse childhood experiences and inflammation

Associations between the three methods of assessing ACEs with inflammation were examined using linear regression. The results are expressed as a percentage difference to aid interpretation given that each inflammatory marker was log-transformed and multiplied by 100 (Cole and Altman, 2017). Two sets of models were fitted for each ACE operationalisation: (i) a crude, unadjusted model, followed by (ii) adjusting for all early life covariates. We also calculated omega-squared (ω^2^) on our covariate-adjusted models to summarise the proportion of variance in inflammation that is explained in the adjusted models. The sample size for the fibrinogen and vWF associations was 8810 and for the CRP n = 7059. All data management, multiple imputation and regression analyses were conducted in Stata version 15.1 (StataCorp, 2017) and the latent class analysis conducted in MPlus version 7.3 (Muthen and Muthen, 2012).

## 3 Results

The composition of the analytic sample on all analysis variables is shown in Table 1. Looking firstly at the prospectively measured ACEs assessed between 1958 and 1974, there was low prevalence of all ACEs amongst NCDS cohort members. The most commonly identified ACE was parental separation/divorce (5.2%), followed by parental offending (4.6%) and parental mental illness (4.3%). The least commonly reported prospective ACE was parental substance misuse (0.6%). The low prevalence of ACEs in the prospectively measured data is reflected in the low ACE scores – most (82.2%) cohort members had no ACEs, 12.9% had 1 ACE and only 5.0% reported 2 or more ACEs. The percentage of cohort members who reported ACEs retrospectively in adulthood was higher, resulting in 60.3% of cohort members with no reported ACEs, 16.2% with 1 ACE, 10.3% with 2 ACEs, 5% with 3 ACEs and 8.3% with 4 or more ACEs. The most commonly reported retrospective ACEs were parental mental illness (25.9%), followed by parental substance misuse (13.5%) and family conflict (13.0%). The least commonly reported was sexual abuse (1.3%).

**Table 1:** Description of the study sample.

N = 8810^a^		%^b^/Median [IQR]	% of missing values^c^
*Outcomes (44/45 years)*			
C-reactive protein (mg/L), median [IQR]		0.9 [0.5, 2.1]	
Fibrinogen (g/L), median [IQR]		2.9 [2.6, 3.2]	
von Willebrand factor (IU, dl), median[IQR]		119 [98, 140]	

*Prospective childhood adversities (0*–*16 years)*			
Parental separation/divorce		5.2	37.3
Parental substance misuse		0.6	33.5
Parental death		2.6	36.4
Parental mental illness		4.3	26.4
Physical neglect		4.4	17.1
Parental offending		4.6	45.4
Family conflict		3.6	33.5
Prospective ACE score			
0 ACEs		82.2	d
1 ACE		12.9	
2 + ACEs		5.0	

*Retrospective childhood adversities (33/44/45 years)*			
Parental separation/divorce		7.5	22.1
Parental substance misuse		13.5	0
Parental mental illness		25.9	0
Family conflict		13.0	0
Emotional neglect		11.4	0
Physical abuse		5.9	0
Sexual abuse		1.3	0
Psychological abuse		9.6	0
Witnessed abuse		5.7	0
Retrospective ACE score			
0 ACEs		60.3	d
1 ACE		16.2	
2 ACEs		10.3	
3 ACEs		5.0	
4 + ACEs		8.3	

*Covariates*			
Gender, % female		50.3	0
Father's social class (birth)			12.7
I professional		5.1	
II managerial & technical		14.5	
IIINM skilled non-manual		10.3	
IIIM skilled manual		50.4	
IV semi-skilled manual		11.8	
V unskilled manual		7.9	
Birthweight (grams), mean (SD)		3342.3 (512.0)	11.9
Maternal age at birth (years), mean (SD)		27.5 (5.6)	8.2
Breastfeeding (reported at 7 years)			17.1
No		29.3	
Up to 1 month		24.3	
Longer than 1 month		46.5	
Mother's education (birth)			8.5
Stayed beyond min leaving age		27.5	
Left before min leaving age		72.5	
Gestational age (days), mean (SD)		281.0 (11.8)	19.1

a Analytic sample is comprised of those with complete information on relevant ACEs in the retrospective ACEs questionnaire at age 44/45 and at least one observed inflammatory marker;

b Pooled percentages are shown as data are imputed and the specific Ns therefore vary across the 20 imputed datasets.

c Missing values reported for those who had complete information on retrospective ACEs questionnaire at age 44/45 and at least one observed inflammatory marker.

d ACE scores were computed post-imputation and therefore have no missing data. Abbreviations: ACE = adverse childhood experience; IQR = interquartile range; SD = standard deviation

### 3.1 The clustering of prospectively measured ACEs in the NCDS

#### 3.1.1 Descriptive clustering of prospectively measured ACEs

Firstly, looking at the prospectively measured ACEs information, Table 2 shows the descriptive co-reporting, expressed as row percentages. This table shows that there is some moderate clustering of ACEs. For example, of those reporting parental substance misuse, more than a third (35.3%) also experienced parental separation/divorce, a third (33.3%) reported parental mental illness, more than 40% reported parental offending and more than three-quarters (78.4%) reported family conflict. The strongest correlations (as represented by the tetrachoric correlation coefficient, rho) were between family conflict and parental substance misuse (0.81); parental separation/divorce and parental death (0.54); parental separation/divorce and family conflict (0.57); and parental offending and parental substance misuse (0.53), although most of these are at a fairly moderate level. Correlations between parental mental illness and physical neglect with all other adversities were low.

**Table 2:** Co-reporting of prospectively measured adversities in the NCDS (row percentages, (Rho)).

	Parental separation/divorce	Parental substance misuse	Parental death	Parental mental illness	Physical neglect	Parental offending	Family conflict
Parental separation/divorce (5.2%)^a^		3.9	16.7	10.8	8.2	20.1	22.5
Parental substance misuse (0.6%)	35.3 (0.45)		7.8	33.3	7.8	41.2	78.4
Parental death (2.6%)	33.2 (0.54)	1.7 (0.19)		10.3	6.5	10.3	12.1
Parental mental illness (4.3%)	13.3 (0.25)	4.5 (0.47)	6.4 (0.21)		8.5	13.6	23.9
Physical neglect (4.4%)	9.8 (0.16)	1.0 (0.10)	3.9 (0.08)	8.3 (0.17)		16.5	12.1
Parental offending (4.6%)	23.2 (0.46)	5.2 (0.53)	6.0 (0.19)	12.7 (0.30)	16.0 (0.37)		23.2
Family conflict (3.6%)	33.1 (0.57)	12.7 (0.81)	8.9 (0.29)	28.7 (0.57)	15.0 (0.33)	29.6 (0.56)	

Rho from tetrachoric correlation only shown in lower part of table as Rho is identical for corresponding pairs of adversities in the upper part ^a^Percentages in parentheses show overall prevalence of each adversity

#### 3.1.2 Latent class analysis of prospectively measured ACEs

Three adversity classes were identified by the LCA on prospectively measured ACEs data (Supplement 2). For each adversity class, the predicted probability of each adversity is shown in Table 3 (left hand pane) and the corresponding profile plot is shown in Supplement 3. The largest class was comprised of cohort members with low probability of reporting any ACE (‘Low ACEs’, 95.7%). 2.8% of cohort members were allocated to the ‘Household dysfunction’ cluster comprised of those who experienced family conflict and to a lesser extent parental mental illness, parental separation and parental offending. The smallest group (1.5% of cohort members) were allocated to the ‘Parental loss’ cluster defined by high probability of experiencing parental separation/divorce and to a lesser extent parental death.

**Table 3:** Predicted probabilities of adversities by class membership.

	Prospective ACE classes			Retrospective ACE classes			
	Low ACEs (95.7%)	Household dysfunction (2.9%)	Parental loss (1.5%)	Low ACEs (76.7%)	Polyadversity (5.2%)	Parental mental illness & substance misuse (12.9%)	Maltreatment & conflict (5.2%)
Parental death	0.02	0.08	0.30				
Physical neglect	0.04	0.21	0.06				
Parental offending	0.03	0.39	0.17				
Parental separation	0.02	0.32	0.91	0.04	0.30	0.11	0.25
Parental substance misuse	0.00	0.14	0.00	0.00	0.72	0.64	0.01
Parental mental illness	0.03	0.38	0.08	0.08	0.98	0.95	0.18
Family conflict	0.01	0.68	0.05	0.02	0.88	0.25	0.51
Sexual abuse				0.00	0.10	0.01	0.08
Physical abuse				0.01	0.53	0.01	0.39
Psychological abuse				0.01	0.79	0.08	0.57
Emotional neglect				0.05	0.51	0.14	0.48
Witnessed abuse				0.00	0.56	0.04	0.28

Abbrevations: ACE = adverse childhood experiences.

#### 3.1.3 Exploring single ACEs, ACE scores and ACEs clusters in associations with adult inflammation based on prospectively measured ACEs data

For individual adversities, parental separation/divorce, physical neglect, parental offending and family conflict were associated with higher CRP levels in mid-life but only the association between parental offending and CRP remained upon covariate adjustment (26.67, 95% CI: 12.11, 43.12) (Table 4). This strong association between parental offending and CRP also remains upon mutual adjustment for all other ACEs (32.40, 95% CI: 16.60, 50.23). Parental separation/divorce, parental death, physical neglect, parental offending and family conflict were also associated with higher levels of fibrinogen in mid-life, and all these associations remained, with the exception of physical neglect, upon covariate adjustment. Parental death (3.77, 95% CI: 1.30, 6.30) and offending (5.36, 95% CI: 3.42, 7.35) showed the strongest associations with fibrinogen. A similar pattern emerged for associations between physical neglect, parental offending and family conflict with vWF. In addition, cohort members who had a parent with a mental illness also had higher vWF levels in mid-life which remained upon adjustment (3.42, 95% CI: 0.19, 6.74). Parental substance misuse was not associated with any of the three inflammatory markers.

**Table 4:** Associations between prospective ACEs measures and mid-life inflammation – individual ACEs, ACE scores and LCA-derived ACE clusters.

	CRP N = 7059		Fibrinogen N = 8810		vWF N = 8810	
	Crude	Adjusted^b^	Crude	Adjusted^b^	Crude	Adjusted^b^
	% difference^a^ (95% CI)	% difference^a^ (95% CI)	% difference^a^ (95% CI)	% difference^a^ (95% CI)	% difference^a^ (95% CI)	% difference^a^ (95% CI)
Parental separation/divorce	13.20 (0.68, 27.27)	7.43 (-4.44, 20.76)	3.23 (1.43, 5.07)	1.95 (0.19, 3.74)	−0.01 (-2.84, 2.92)	−0.22 (-3.06, 2.70)
Parental substance misuse	−19.50 (−42.20, 12.13)	−24.26 (−45.49, 5.23)	4.27 (−0.99, 9.81)	2.70 (−2.41, 8.07)	3.68 (−4.73, 12.82)	2.32 (−5.96, 11.34)
Parental death	11.99 (−4.99, 32.00)	8.97 (−7.43, 28.28)	4.46 (1.93, 7.05)	3.77 (1.30, 6.30)	3.14 (−0.91, 7.35)	2.48 (−1.53, 6.66)
Parental mental illness	8.25 (−4.89, 23.22)	4.16 (−8.39, 18.44)	1.65 (−0.30, 3.65)	0.86 (−1.05, 2.80)	3.91 (0.66, 7.26)	3.42 (0.19, 6.74)
Physical neglect	18.40 (4.22, 34.51)	10.51 (−2.69, 25.51)	2.77 (0.82, 4.75)	1.62 (−0.28, 3.57)	5.03 (1.79, 8.36)	3.64 (0.43, 6.94)
Parental offending	35.95 (20.30, 53.63)	26.67 (12.11, 43.12)	6.03 (4.06, 8.05)	5.36 (3.42, 7.35)	5.57 (2.38, 8.87)	3.99 (0.83, 7.26)
Family conflict	11.90 (4.10, 20.28)	11.95 (−2.54, 28.58)	2.52 (1.40, 3.64)	1.59 (0.50, 2.67)	1.94 (0.14, 3.77)	1.75 (−0.05, 3.58)
ACE score						
0	Ref	Ref	Ref	Ref	Ref	Ref
1	19.38 (10.54, 28.94)	13.72 (5.31, 22.80)	2.67 (1.47, 3.88)	1.92 (0.74, 3.11)	2.90 (0.94, 4.89)	2.09 (0.14, 4.07)
2+	23.90 (9.84, 39.76)	15.89 (2.78, 30.69)	5.14 (3.25, 7.06)	3.90 (2.05, 5.77)	4.89 (1.83, 8.04)	3.86 (0.82, 6.99)
LCA-derived ACE clusters						
Low ACEs	Ref	Ref	Ref	Ref	Ref	Ref
Household dysfunction	22.97 (4.91, 44.14)	15.61 (−1.29, 35.41)	4.42 (1.96, 6.93)	3.11 (0.72, 5.56)	5.64 (1.61, 9.82)	4.55 (0.56, 8.70)
Parental loss	0.82 (−18.93, 25.40)	−4.52 (−23.10, 18.55)	4.74 (1.37, 8.22)	3.52 (0.25, 6.91)	2.60 (−2.73, 8.23)	2.26 (−3.05, 7.85)

a Results are expressed as percentage difference in the inflammatory marker to aid interpretation as each marker was log-transformed and multiplied by 100 due to positive skew.

b Model is adjusted for birthweight, gestational age, gender, father’s social class at birth, mother’s education and breastfeeding duration. Abbrevations: ACE(s) = Adverse Childhood Experience(s); CRP = C-Reactive Protein; LCA = Latent Class Analysis; vWF = von Willebrand Factor.

A graded association between the number of ACEs reported and all three markers of inflammation was observed. This graded association remained upon adjustment for early life factors; cohort members experiencing 2 or more ACEs had 15.89% higher CRP (95% CI: 2.78, 30.69), 3.90% higher fibrinogen (95% CI: 2.05, 5.77) and 3.86% higher vWF (95% CI: 0.82, 6.99) compared to those reporting no ACEs.

Finally, for ACE classes, those in the ‘Household dysfunction’ class had higher levels of CRP (22.97, 95% CI: 4.91, 44.14), fibrinogen (4.42, 95% CI: 1.96, 6.93) and vWF (5.64, 95% CI: 1.61, 9.82) compared to those in the ‘Low ACEs’ group, but this was no longer statistically significant at the 0.05 level upon adjustment in the case of CRP. Cohort members in the ‘Parental loss’ group also had higher levels of fibrinogen in mid-life compared to those in the ‘Low ACEs’ group and this remained in the adjusted model (3.52, 95% CI: 0.25, 6.91).

Examining the three ACEs operationalisations by the proportion of variance explained (Supplement 4), parental offending as a singly-considered ACE and ACE score had the highest ω^2^ values across all three inflammatory markers. This further reflects the findings reported above.

### 3.2 The clustering of retrospectively reported ACEs in the NCDS

#### 3.2.1 Descriptive clustering of retrospectively reported ACEs

Generally, stronger clustering was seen for retrospectively reported ACEs compared to prospectively reported ACEs (Table 5). For instance, of those reporting parental substance misuse, 16.6% reported parental separation/divorce, nearly all reported parental mental illness (93.8%), 41.8% reported family conflict, around a quarter reported emotional neglect (24.2%) or psychological abuse (25.9%), 15.8% reported physical abuse and 19.5% reported witnessing abuse. In these retrospective data the strongest correlations were between parental mental illness and parental substance misuse (rho = 0.91), physical and psychological abuse (0.85), physical abuse and witnessing abuse (0.79), psychological abuse and family conflict (0.77), and psychological abuse and witnessing abuse (0.74).

**Table 5:** Co-reporting of retrospectively reported adversities in the NCDS (row percentages, (Rho)).

	Parental separation/divorce	Parental substance misuse	Parental mental illness	Family conflict	Emotional neglect	Psychological abuse	Physical abuse	Sexual abuse	Witnessed abuse
Parental separation/divorce (7.5%)^a^		30.0	47.4	42.4	31.1	26.8	19.2	6.7	19.2
Parental substance misuse (13.5%)	16.6 (0.35)		93.8	41.8	24.2	25.9	15.8	3.5	19.5
Parental mental illness (25.9%)	13.7 (0.31)	49.0 (0.91)		33.5	20.8	23.1	12.8	2.9	14.1
Family conflict 13.0%)	24.3 (0.54)	43.4 (0.59)	66.6 (0.62)		37.8	45.9	27.2	5.7	29.8
Emotional neglect (11.4%)	20.3 (0.41)	28.7 (0.33)	47.2 (0.34)	43.1 (0.58)		36.7	23.9	4.4	19.9
Psychological abuse (9.6%)	21.0 (0.42)	36.8 (0.45)	62.9 (0.53)	62.7 (0.77)	44.0 (0.61)		45.1	8.4	35.2
Physical abuse (5.9%)	24.4 (0.42)	36.4 (0.39)	56.6 (0.40)	60.3 (0.67)	46.5 (0.57)	73.3 (0.85)		10.7	48.6
Sexual abuse (1.3%)	38.6 (0.45)	36.0 (0.29)	57.0 (0.32)	57.0 (0.50)	38.6 (0.36)	61.4 (0.60)	48.3 (0.60)		50.9
Witnessed abuse (5.7%)	25.2 (0.44)	46.3 (0.51)	64.3 (0.49)	68.1 (0.73)	39.9 (0.49)	58.9 (0.74)	50.1 (0.79)	11.6 (0.62)	

Rho from tetrachoric correlation only shown in lower part of table as Rho is identical for corresponding pairs of adversities in the upper part ^a^Percentages in parentheses show overall prevalence of each adversity

#### 3.2.2 Latent class analysis of retrospectively reported ACEs

The 4 class solution was decided upon for the retrospective ACEs data as this was the class solution beyond which there were limited returns in terms of the BIC value (Supplement 5). For each ACEs class, the predicted probability of each adversity is shown in the right hand pane of Table 3 and the corresponding profile plot is shown in Supplement 6. Similar to the prospective data, the largest group was the ‘Low ACEs’ cluster (76.7%) comprised of cohort members with low probability of reporting any ACEs. This was a smaller group than in the prospective data, reflecting the higher prevalence and number of ACEs in the retrospective data. Next there was a cluster comprised of cohort members reporting ‘Parental mental illness and substance misuse’ (12.9%). Finally, there were two smaller groups each comprised of 5.2% of cohort members. The first was ‘Polyadversity’ containing cohort members who reported parental substance misuse, parental mental illness, family conflict, psychological abuse and to a more moderate extent - physical, emotional and witnessing abuse. The other group was made up of cohort members reporting family conflict, psychological abuse and emotional neglect (‘Maltreatment and conflict’).

#### 3.2.3 Examining single ACEs, ACE scores and ACEs clusters in associations with adult inflammation based on retrospectively reported ACEs data

The associations between each ACE operationalisation method and mid-life inflammation based on retrospectively reported ACEs data are shown in Table 6. Looking firstly at the individual ACEs, parental separation/divorce was associated with higher CRP and fibrinogen but this did not remain upon adjustment. Family conflict and psychological abuse were both associated with higher CRP (family conflict: 9.05, 95% CI: 0.96, 17.78; psychological abuse: 9.51, 95% CI: 0.17, 19.73) and fibrinogen (family conflict: 1.73, 95% CI: 0.56, 5.02; psychological abuse: 2.33, 95% CI: 0.99, 3.69) in the adjusted models. Similarly, emotional neglect was associated with higher levels of fibrinogen and vWF which remained upon adjustment for covariates. A strong association between physical abuse and all three inflammatory markers was observed; those reporting physical abuse in childhood had 17.57% higher levels of CRP (95% CI: 5.27, 31.31), 3.42% higher levels of fibrinogen (95% CI: 1.73, 5.14) and 4.72% higher levels of vWF (95% CI: 1.90, 7.62) compared to those not reporting physical abuse. These associations also remained upon mutual adjustment for all other adversities (CRP: 14.53, 95% CI: −0.44, 31.76; fibrinogen: 2.91, 95% CI: 0.76, 5.10; vWF: 5.18, 95% CI: 1.62, 8.87). Cohort members reporting witnessing abuse also had higher levels of CRP at age 44/45 (13.75, 95% CI: 1.90, 26.98). Retrospective reports of parental substance misuse, parental mental illness and sexual abuse were not associated with inflammation.

**Table 6:** Associations between retrospective ACEs measures and mid-life inflammation – individual ACEs, ACE scores and LCA-derived ACE clusters.

	CRP N = 7059		Fibrinogen N = 8810		vWF N = 8810	
	Crude	Adjusted^b^	Crude	Adjusted^b^	Crude	Adjusted^b^
	% difference^a^ (95% CI)	% difference^a^ (95% CI)	% difference^a^ (95% CI)	% difference^a^ (95% CI)	% difference^a^ (95% CI)	% difference^a^ (95% CI)
Parental separation/divorce	12.54 (1.95, 24.23)	8.57 (−1.68, 19.88)	2.31 (0.79, 3.85)	1.36 (−0.13, 2.88)	0.75 (−1.69, 3.24)	0.83 (−1.63, 3.34)
Parental substance misuse	5.94 (−1.76, 14.26)	4.22 (−3.32, 12.34)	0.92 (−0.24, 2.08)	0.23 (−0.90, 1.37)	0.35 (−1.51, 2.26)	0.10 (−1.76, 2.00)
Parental mental illness	−2.78 (−8.35, 3.13)	−4.29 (−9.74, 1.49)	0.03 (−0.86, 0.93)	−0.58 (−1.45, 0.31)	−0.55 (−2.00, 0.92)	−0.61 (−2.05, 0.86)
Family conflict	11.38 (3.08, 20.35)	9.05 (0.96, 17.78)	2.62 (1.43, 3.82)	1.73 (0.56, 5.02)	1.82 (−0.11, 3.78)	1.80 (−0.13, 3.76)
Emotional neglect	7.12 (−1.19, 16.12)	4.10 (−3.92, 12.78)	1.83 (0.58, 3.09)	1.28 (0.06, 2.52)	3.35 (1.29, 5.45)	2.87 (0.82, 4.96)
Psychological abuse	12.02 (2.42, 22.52)	9.51 (0.17, 19.73)	3.11 (1.74, 4.50)	2.33 (0.99, 3.69)	1.87 (−0.33, 4.12)	1.88 (−0.32, 4.14)
Physical abuse	22.02 (9.18, 36.36)	17.57 (5.27, 31.31)	4.21 (2.49, 5.97)	3.42 (1.73, 5.14)	5.14 (2.31, 8.05)	4.72 (1.90, 7.62)
Sexual abuse	−2.63 (−22.43, 22.22)	−9.32 (−27.71, 13.76)	1.90 (−1.58, 5.50)	−1.07 (−4.41, 2.38)	2.88 (−2.80, 8.89)	3.03 (−2.67, 9.06)
Witnessed abuse	19.29 (6.82, 33.22)	13.75 (1.90, 26.98)	1.75 (0.04, 3.49)	0.35 (−1.31, 2.05)	1.69 (−1.09, 4.54)	1.25 (−1.52, 4.09)

	**CRP N = 7059**		**Fibrinogen N = 8810**		**vWF n = 8810**	

	**Crude**	**Adjusted**^b^	**Crude**	**Adjusted**^b^	**Crude**	**Adjusted**^b^

	**% difference**^a^**(95% CI)**	**% difference (95% CI)**	**% difference (95% CI)**	**% difference (95% CI)**	**% difference (95% CI)**	**% difference (95% CI)**

ACE score						
0	Ref	Ref	Ref	Ref	Ref	Ref
1	−4.42 (−11.07, 2.69)	−5.83 (−12.30, 1.12)	−0.61 (−1.70, 0.48)	−1.02 (−2.08, 0.06)	−0.01 (−1.79, 1.80)	−0.05 (−1.82, 1.75)
2	4.93 (−3.88, 14.54)	3.45 (−5.16, 12.85)	1.05 (−0.27, 2.40)	0.67 (−0.63, 1.98)	−0.83 (−2.94, 1.34)	−0.91 (−3.02, 1.24)
3	6.82 (−5.54, 20.81)	3.23 (−8.65, 16.66)	−0.65 (−2.45, 1.19)	−1.57 (−3.33, 0.22)	−0.11 (−3.06, 2.92)	−0.09 (−3.03, 2.95)
4+	12.14 (1.93, 23.37)	7.66 (−2.12, 18.42)	3.55 (2.05, 5.06)	2.15 (0.69, 3.64)	3.05 (0.63, 5.54)	2.75 (0.32, 5.23)
LCA-derived ACE clusters						
Low ACEs	Ref	Ref	Ref	Ref	Ref	Ref
Polyadversity	10.88 (−1.42, 24.73)	7.71 (−4.19, 21.09)	4.82 (2.97, 6.70)	3.51 (1.71, 5.34)	3.11 (0.16, 6.16)	2.86 (−0.09, 5.90)
Parental mental illness & substance misuse	3.53 (−4.24, 11.93)	1.81 (−5.77, 10.01)	−0.18 (−1.35, 6.70)	−0.72 (−1.83, 0.44)	−0.88 (−2.77, 1.05)	−0.93 (−2.81, 1.00)
Maltreatment & conflict	19.78 (6.32, 34.95)	15.30 (2.41, 29.82)	3.28 (1.45, 5.13)	2.47 (0.69, 4.29)	1.13 (−1.78, 4.12)	0.87 (−2.02, 3.86)

a Results are expressed as percentage difference in the inflammatory marker to aid interpretation as each marker was log-transformed and multiplied by 100 due to positive skew.

b Model is adjusted for birthweight, gestational age, gender, father’s social class at birth, mother’s education and breastfeeding duration. Abbrevations: ACE(s) = Adverse Childhood Experience(s); CRP = C-Reactive Protein; LCA = Latent Class Analysis; vWF = von Willebrand Factor

Next looking at the associations between cumulative ACE score and inflammation, cohort members who reported 4 or more ACEs had higher levels of all three inflammatory markers with associations with fibrinogen and vWF remaining upon covariate adjustment (fibrinogen: 2.15, 95% CI: 0.69, 3.64; vWF: 2.75, 95% CI: 0.32, 5.23). Finally, looking at associations between the LCA-derived adversity clusters and inflammation, compared to those in the ‘Low ACEs’ cluster, those in the ‘Polyadversity’ cluster had higher levels of fibrinogen (3.51, 95% CI: 2.97, 6.70) and vWF (2.86, 95% CI: −0.09, 5.90). Furthermore, those in the ‘Maltreatment and conflict’ cluster had higher levels of CRP (15.30, 95% CI: 2.41, 29.82) and fibrinogen (2.47, 95% CI: 0.69, 4.29) relative to people in the ‘Low ACEs’ group. Cohort members in the ‘Parental mental illness and substance misuse’ class did not have higher levels of inflammation compared to the ‘Low ACEs’ cluster. As expected, the associations between LCA-derived clusters and inflammation reflected those seen for the individual ACE items. Additionally, the results for the ‘Polyadversity’ group are also similar for associations between an ACE score of 4+ for vWF and fibrinogen, although the LCA-derived clusters explained more of the variance in fibrinogen than other operationalisations of ACEs (Supplement 7). The magnitude of the ω^2^ values again reflected the strengths of the associations observed in Table 6, with the highest ω^2^ values for CRP and vWF seen for physical abuse, and the highest value for fibrinogen being the LCA-derived classes followed by physical abuse.

## 4 Discussion

### 4.1 Summary of main findings

Using data from a large longitudinal study – the 1958 British birth cohort - we observed a moderate level of clustering in the prospective ACEs data which reflected the low prevalence of ACEs reported prospectively. However, a strong correlation was observed between family conflict and parental substance misuse. Applying LCA to the prospective ACEs data, cohort members in the ‘Parental loss’ group had higher levels of fibrinogen in mid-life compared to those in the ‘Low ACEs’ group. Furthermore, those in the ‘Household dysfunction’ class had higher fibrinogen and vWF compared to the ‘Low ACEs’ group. Whilst the LCA appeared to work well, at least empirically, the two adversity groups were largely driven by one item each – family conflict for the ‘Household dysfunction’ class and parental separation/divorce for the ‘Parental loss’ group. Consequently, the findings for the LCA-derived clusters and these individual ACEs largely mirrored one another. In contrast, a graded association was seen between the prospective ACE score and all three inflammatory markers. In terms of the proportion of variance explained by the different ACE operationalisations, parental offending as an individual ACE and the ACE score contributed the most.

The level of clustering in the retrospectively reported ACEs data was much higher than observed in the prospectively measured information and most of the correlation coefficients were >0.7. The correlation between parental mental illness and substance misuse was particularly high. Unlike in the prospective LCA, the classes were comprised of cohort members reporting multiple adversities. The ‘Maltreatment and conflict’ group was associated with higher CRP and fibrinogen in mid-life, and the ‘Polyadversity’ group was associated with higher fibrinogen and vWF. Family conflict, emotional neglect, psychological abuse, physical abuse and the witnessing of abuse were all associated with at least one inflammatory marker in covariate-adjusted models. Only those with an ACE score of 4+ had higher levels of fibrinogen or vWF. With respect to the omega-squared values, physical abuse, emotional neglect and the LCA-derived classes of ‘Polyadversity’ and ‘Maltreatment and conflict’ (for fibrinogen) explained the largest proportions of the variance in inflammation.

### 4.2 Interpretation of study findings

The low prevalence of ACEs reported prospectively might be due to social desirability bias, particularly for adversities like parental mental illness and the reducing stigma and increasing acceptability of reporting mental health problems over time (these data were reported from 1958 to 1974). The low prevalence of prospectively measured ACEs is reflected also in the low correlations between adversities in those data – ACEs with higher prevalence are more likely to co-occur with other ACEs. The differences in the strength of clustering between the prospective and retrospective ACEs reports might be explained by common method variance. All retrospective ACEs, except parental separation/divorce which was also reported at age 33, were collected via the same questionnaire at age 44/45. In contrast, the prospective data was collected from parents, health visitors and teachers at ages 7, 11 and 16. Common method variance can artificially inflate the strength of relationship between ACEs (Schaller et al., 2015). There has been little discussion of the impact of common method variance in the ACEs literature, despite the widespread use of retrospective ACE questionnaires or checklists. This is something that should be considered in future work as the correlations between ACEs could be an artefact of the method of data collection. New methods are emerging which aid the detection and correction for common method variance (Tehseen et al., 2017)

Parental separation/divorce and death, reported prospectively, were associated with higher fibrinogen in mid-life and this is consistent with previous work in this cohort (Lacey et al., 2013). Parental offending showed the strongest association with all three inflammatory markers over and above all other prospectively measured adversities, and this may be due to this being a very public adversity which was likely to cause shame and stigma at the time, resulting in a potentially greater stress response. The relationship between parental incarceration and higher CRP levels was previously found in the US National Longitudinal Study of Adolescent to Adult Health (Boch and Ford, 2014), however it was not previously compared to other adversities. This finding suggests that it might be important to support families, and particularly children, who experience parental offending in particular. Similarly, in the retrospective data physical abuse and other ‘threat-based’ adversities, such as witnessing abuse and family conflict were associated with higher levels of inflammation. This is consistent with theoretical work by McLaughlin et al. (2014) which suggests that threat-based adversities are more likely to activate the HPA axis and consequently result in chronic inflammation. Previous work on the 1958 cohort also showed higher inflammation for those reporting physical abuse (Pinto Pereira et al., 2019). In contrast, a recent study showed that official reports, but not retrospective self-reports, of physical abuse were associated with higher CRP (Osborn and Widom, 2019). This, in combination, with our study suggests that the retrospective and prospective reports show different associations with inflammation.

The LCA didn’t work as well as expected in the prospective data and this is likely driven by the low prevalence and consequently low correlations between prospectively measured ACEs. The findings from the LCA method and individual adversities largely mirrored one another. These findings suggest that in contexts where there are low correlations between variables the LCA doesn’t offer much over and above looking at individual ACEs. However, before discounting LCA this should be tested on other prospective datasets where more adversities are available. There was a graded association between prospectively reported ACE scores and all three markers of inflammation, which has been observed previously in this cohort (Chen and Lacey, 2018). However it has been argued that ACE scores are only appropriate to use where the adversities included correlate highly with one another (Evans et al., 2013) and as such may be inappropriate in this context.

In contrast, the LCA appeared to be more successful in the retrospective data. Four classes were identified and these were consistent with those found in other work. For instance, the ‘Low ACEs’ and ‘Polyadversity’ classes (sometimes labelled ‘High ACEs’ or ‘High adversity’) are found in many studies applying LCA to ACEs data (Cavanaugh et al., 2015, Debowska et al., 2017, Lanier et al., 2018, Rapsey et al., 2018, Salas et al., 2019). The ‘Polyadversity’ group was associated with the highest levels of fibrinogen and vWF on average, suggesting that people reporting many adversities might be vulnerable to chronic inflammation, and this finding is also reflected in higher levels of inflammation for the highest ACE score category results. Past studies showed that a ‘Polyadversity’ class had the highest risk of negative outcomes, including post-traumatic stress disorder (Ford et al., 2010), personality disorder (Charak et al., 2018), internalising disorders (Rapsey et al., 2018) and eating disorder symptoms (Hazzard et al., 2019). The ‘Maltreatment and conflict’ group also had the highest increase in CRP levels, on average, and also had higher fibrinogen levels compared to the ‘Low ACEs’ group. This finding suggests that the combination of family conflict, psychological abuse and emotional neglect might be particularly risky for chronic inflammation. Most previous studies have only applied LCA to child maltreatment variables. This study represents an extension of this work to broader ACEs, such as parental mental illness, substance misuse and parental separation/divorce, as well as to a British population and to inflammatory outcomes.

### 4.3 Methodological considerations

#### 4.3.1 Limitations

The LCA approach to ACEs operationalisation is not without its limitations. The findings from LCA might be dataset specific given that is a data driven approach. Given that there is currently no consensus on the number or nature of classes, we followed Debowska et al. (2017) recommendation of an exploratory rather than confirmatory approach. The LCA approach can also be difficult to interpret for users of ACEs research (Debowska et al., 2017), however we provided profile plots to aid interpretation as recommended. We dropped cohort members who had CRP values ≥10 mg/L from our analyses. No such cut-points exist for vWF or fibrinogen. We conducted sensitivity analyses restricting the sample for vWF and fibrinogen associations to those in the CRP sample (n = 7059). This did not alter the associations observed for these two inflammatory markers (results not shown). Finally, whilst the NCDS has rich prospective and retrospective ACEs information, the prospective data referred to 1958–1974. The prevalence of all of the prospectively measured ACEs data is low, and certainly lower than in more recent birth cohorts (Houtepen et al., 2018). This might have been affected by social desirability bias. However, whilst some might consider the NCDS a historical cohort, it enables us to take a long-term view of how ACEs might affect inflammation in mid-life.

#### 4.3.2 Strengths

The strengths of the present study are the use of a large, prospective birth cohort study which enabled us to explore the long-term relationships between ACEs and mid-life inflammation. The sample is broadly representative of British men and women of a similar age (Atherton et al., 2008). We minimised potential bias attributable to missing data by applying multiple imputation. We also had rich ACEs information reported prospectively and retrospectively. Finally, the longitudinal design also allowed us to take account of potential early life confounders captured at birth, prior to experience of ACEs.

## 5 Conclusions

Using a large, longitudinal birth cohort with prospective and retrospective information on ACEs we tested three methods of operationalising ACEs – single ACEs, ACE scores and LCA-derived ACE clusters – and their associations with inflammation in mid-life. We found a higher level of correlation between retrospectively-reported ACEs than prospectively-collected ACEs. Further research is needed to disentangle whether there is indeed real co-reporting of ACEs or whether this is due to common method variance due to collection of multiple ACEs from a single questionnaire. The assessment of common method variance should be considered in future ACEs research which employs a single questionnaire to collect retrospectively-reported information. Conversely, in situations where there is low correlation between ACEs – such as in our prospective ACEs data – LCA may not offer additional information than considering individual ACEs.

LCA might offer an alternative approach for operationalising ACEs where there is co-occurrence and little potential for common method variance. The LCA did not explain more of the variance in the prospectively-collected ACEs data where there was less co-occurrence of ACEs. In conclusion, we found that parental offending, physical abuse, family conflict and emotional neglect showed the strongest associations with inflammation. If assumed to be causal, these findings suggest that parental offending, physical abuse and emotional neglect are particular risk factors for higher inflammatory levels in mid-life.
