# Supplementary material for: Adverse childhood experiences and adult inflammation: Single adversity, cumulative risk and latent class approaches
Source: Brain Behav Immun. 2020 Jul;87:820–30. doi: 10.1016/j.bbi.2020.03.017 (PMC7327510; doi:10.1016/j.bbi.2020.03.017)
Supplement: Supplementary data 1 [file mmc1.docx]

**Supplementary material**

Supplement 1 Further information on adverse childhood experience variables used in the NCDS

| **Adversity** | **Age collected** | **Informant** | **Description** |
| --- | --- | --- | --- |
| **Prospectively measured ACEs** | | | |
| Parental separation/divorce | 7 | Health visitor | Divorce or separation listed as difficulty of the family |
|  | 11, 16 | Parent | Relationship to child of person acting as child's parents & reason for change |
| Parental substance misuse | 7 | Health visitor | Alcoholism listed as difficulty of the family |
| Family conflict | 7 | Health visitor | Domestic tension listed as difficulty of the family |
| Death of parent | 7 | Health visitor | Death of mother listed as difficulty of the family |
|  | 7 | Health visitor | Death of child's father listed as difficulty of the family |
|  | 11, 16 | Parent | Relationship to child of person acting as child's parents & reason for change |
| Parental mental health problems | 7 | Health visitor | Mental illness or neurosis listed as difficulty of the family |
|  | 7 | Health visitor | Family uses services of a psychiatric social worker |
|  | 7, 11, 16 | Parent | Mother or father has a chronic mental illness |
| Physical neglect | 7, 11 | Teacher | Child appears scruffy/dirty/underfed |
| Parental offending | 7 | Health visitor | Family in contact with probation services |
|  | 11 | Parent | Family in contact with probation services or family member in prison |
|  | 16 | Parent | Family member in contact with probation services |
| **Retrospectively reported ACEs** | | | |
| Parental separation/divorce | 33 | Cohort member | Parents ever permanently separated or divorced & how old when happened |
| Parental substance misuse | 44/45 | Cohort member | Mother had trouble with drinking or other drug use |
|  | 44/45 | Cohort member | Father had trouble with drinking or other drug use |
| Family conflict | 44/45 | Cohort member | There was much conflict and tension in the household whilst I was growing up |
| Witnessing abuse | 44/45 | Cohort member | I witnessed physical or sexual abuse of others in my family |
| Parental mental health problems | 44/45 | Cohort member | Mother or father suffered from nervous or emotional trouble or depression |
| Sexual abuse | 44/45 | Cohort member | I was sexually abused by a parent |
| Physical abuse | 44/45 | Cohort member | I was physically abused by a parent - punched, kicked or hit or beaten with an objective, or needed medical treatment |
| Psychological abuse | 44/45 | Cohort member | I was verbally abused by a parent |
|  | 44/45 | Cohort member | I suffered humiliation, ridicule, bullying or mental cruelty from a parent |
| Emotional neglect | 44/45 | Cohort member | My mother was unaffectionate |
|  | 44/45 | Cohort member | My father was unaffectionate |

Abbreviations: ACEs = adverse childhood experiences

NB Where more than one variable was used to indicate each adversity, cohort members responding positively to either were recorded as having reported/experienced that adversity

Supplement 2 Comparing latent class analysis solutions on prospectively measured ACEs data

|  | **6 class solution** | **5 class solution** | **4 class solution** | **3 class solution** | **2 class solution** |
| --- | --- | --- | --- | --- | --- |
| **N (%) cohort members in each class** | |  |  |  |  |
| Class 1 | 66 (0.1) | 57 (0.6) | 394 (3.3) | **8434 (95.7)** | 83321 (94.5) |
| Class 2 | 95 (1.1) | 8154 (92.6) | 104 (1.2) | **247 (2.8)** | 489 (5.6) |
| Class 3 | 392 (4.4) | 401 (4.6) | 8062 (91.5) | **129 (1.5)** |  |
| Class 4 | 8154 (92.6) | 79 (0.9) | 250 (2.8) |  |  |
| Class 5 | 40 (0.5) | 119 (1.4) |  |  |  |
| Class 6 | 63 (0.7) |  |  |  |  |
| **Model fit indices** |  |  |  |  |  |
| BIC | 17670.6 | 17617 | 17584.4 | **17558.6** | 17589.2 |
| SSABIC | 17521.2 | 17493.1 | 17485.9 | **17485.5** | 17541.5 |
| AIC | 17337.6 | 17340.8 | 17364.8 | **17395.7** | 17589.2 |
| Entropy | 0.92 | 0.89 | 0.85 | **0.92** | 0.85 |

Abbreviations: AIC = Akaike Information Criterion; BIC = Bayesian Information Criterion; SSABIC = Sample Size Adjusted Bayesian Information Criterion

Supplement 3 Profile plot of adversities by class membership in the three-class solution for prospectively measured ACEs data

Abbreviations: ACEs = Adverse Childhood Experiences

Supplement 4 Omega-squared (ω^2^) values for associations between prospective ACEs and mid-life inflammation shown in table 4

|  | **CRP**  **ω^2^** | **Fibrinogen**  **ω^2^** | **vWF**  **ω^2^** |
| --- | --- | --- | --- |
| Parental separation/divorce | 2.04 | 3.61 | 0.84 |
| Parental substance misuse | 2.06 | 3.57 | 0.85 |
| Parental death | 2.04 | 3.66 | 0.86 |
| Parental mental illness | 2.03 | 3.57 | 0.89 |
| Physical neglect | 2.06 | 3.59 | 0.90 |
| Parental offending | **2.22** | **3.89** | **0.91** |
| Family conflict | 2.06 | 3.65 | 0.89 |
| ACE score | **2.22** | **3.84** | **0.93** |
| LCA-derived ACE clusters | 2.06 | 3.67 | 0.90 |

Abbreviations: ACE = Adverse Childhood Experience; CRP = C-Reactive Protein; LCA = Latent Class Analysis; vWF = von Willebrand Factor

Supplement 5 Comparing latent class analysis solutions on retrospectively reported ACEs data

|  | **6 class solution** | **5 class solution** | **4 class solution** | **3 class solution** | **2 class solution** |
| --- | --- | --- | --- | --- | --- |
| **N (%) participants in each class** | |  |  |  |  |
| Class 1 | 531 (6.0) | 380 (4.3) | **6760 (76.7)** | 842 (9.6) | 2013 (22.8) |
| Class 2 | 5810 (65.9) | 215 (2.4) | **457 (5.2)** | 1012 (11.5) | 6797 (77.2) |
| Class 3 | 225 (2.6) | 899 (10.2) | **1139 (12.9)** | 6956 (79.0) |  |
| Class 4 | 505 (5.7) | 6544 (74.3) | **454 (5.2)** |  |  |
| Class 5 | 1533 (17.4) | 772 (8.8) |  |  |  |
| Class 6 | 206 (2.3) |  |  |  |  |
| **Model fit indices** |  |  |  |  |  |
| BIC | 39570.2 | 39589 | **39668.9** | 40026.6 | 41644.7 |
| SSABIC | 39382.7 | 39433.3 | **39545.0** | 39934.5 | 41584.3 |
| AIC | 39152.2 | 39241.9 | **39392.6** | 39821.2 | 41510.1 |
| Entropy | 0.83 | 0.84 | **0.87** | 0.88 | 0.85 |

Abbreviations: AIC = Akaike Information Criterion; BIC = Bayesian Information Criterion; SSABIC = Sample Size Adjusted Bayesian Information Criterion

Supplement 6 Profile plot of adversities by class membership in the four class solution for retrospectively-reported ACEs data

Abbreviations: ACE = Adverse Childhood Experiences

Supplement 7 Omega-squared (ω^2^) values for associations between retrospective ACEs and mid-life inflammation shown in table 6

|  | **CRP**  **ω^2^** | **Fibrinogen**  **ω^2^** | **vWF**  **ω^2^** |
| --- | --- | --- | --- |
| Parental separation/divorce | 2.06 | 3.60 | 0.85 |
| Parental substance misuse | 2.04 | 3.56 | 0.84 |
| Parental mental illness | 2.05 | 3.58 | 0.85 |
| Family conflict | 2.09 | 3.66 | 0.88 |
| Emotional neglect | 2.04 | 3.61 | **0.93** |
| Psychological abuse | 2.08 | 3.69 | 0.87 |
| Physical abuse | **2.14** | **3.74** | **0.97** |
| Sexual abuse | 2.03 | 3.57 | 0.86 |
| Witnessed abuse | 2.10 | 3.56 | 0.85 |
| ACE score | 2.08 | 3.73 | 0.88 |
| LCA-derived ACE clusters | 2.09 | **3.81** | 0.88 |

Abbreviations: ACE = Adverse Childhood Experience; CRP = C-Reactive Protein; LCA = Latent Class Analysis; vWF = von Willebrand Factor
